# Supplementary material for: Estradiol elicits distinct firing patterns in arcuate nucleus kisspeptin neurons of females through altering ion channel conductances
Source: bioRxiv. 2024 Sep 3:2024.02.20.581121. Preprint. [Version 3] doi: 10.1101/2024.02.20.581121 (PMC11195100; doi:10.1101/2024.02.20.581121)
Supplement: Supplement 1 [file NIHPP2024.02.20.581121v3-supplement-1.pdf]

bioRxiv preprint doi: https://doi.org/10.1101/2024.02.20.581121; this version posted September 3, 2024. The copyright holder for this preprint (which was not certified by peer review) is the author/funder, who has granted bioRxiv a license to display the preprint in perpetuity. It is made available under aCC-BY 4.0 International license.

Supplemental Information:

A mathematical model of the arcuate nucleus kisspeptin neuron

A schematic diagram of the Arcuate nucleus Kiss1 ($Kiss1^{ARH}$) neuron model is presented in Fig. S1 and parameter values used in the simulations are given in Table S1.

The equation describing the membrane potential, V_m, of $Kiss1^{ARH}$ neurons is given by

$$C_m \frac{dV_m}{dt} = -I,$$

where C_m is the membrane capacitance and I is the sum of 12 ionic currents:

$$I = I_{NaT} + I_{NaP} + I_A + I_{BK} + I_h + I_{SK} + I_M + I_T + I_{Ca} + I_{TRPC5} + I_{GIRK} + I_{leak}$$

I_{NaT} and I_{NaP} are the transient and persistent sodium currents, respectively; I_A represents the A current; I_M represents the M current; I_{SK} and I_{BK} are the potassium currents through the SK and BK channels respectively; I_T the T-type calcium current; I_{Ca} represents other calcium currents (L-, N-, P/Q-, and R-type); I_{TRPC5} represents Calcium current through the TRPC5 channel; I_{GIRK} potassium current through the GIRK channels. Finally, I_{leak} represents the contribution of leak currents.

We use the Hodgkin-Huxley formalism to model current dynamics and their dependence on the membrane potential. Below we detail are the equations governing the currents.

Transient sodium current

$$I_{NaT} = g_{NaT} \cdot m_{NaT,\infty}(V_m) \cdot h_{NaT} \cdot (V_m - E_{Na}),$$

where g_{NaT} is the maximum conductance; E_{Na} is the sodium reversal potential; h_{NaT} is the inactivation gating variable that obeys the following equation:

$$\frac{dh_{NaT}}{dt} = \frac{h_{NaT,\infty}(V_m) - h_{NaT}}{\tau_{h,NaT}}.$$

Parameter $\tau_{h,NaT}$ dictates the timescale of inactivation and $h_{NaT,\infty}(V_m)$ is the steady-state inactivation function:

$$h_{NaT,\infty}(V_m) = \frac{1}{1+e^{-(V_m - V_{h,NaT})/k_{h,NaT}}}.$$

Parameter $V_{h,NaT}$ describes the voltage achieving half-maximal inactivation and parameter $k_{h,NaT}$ is the associated scaling function.

Finally, in the current formulation $m_{NaT,\infty}(V_m)$ is the steady-state activation function given by:

$$m_{NaT,\infty}(V_m) = \frac{1}{1+e^{-(V_m - V_{m,NaT})/k_{m,NaT}}}.$$

bioRxiv preprint doi: https://doi.org/10.1101/2024.02.20.581121; this version posted September 3, 2024. The copyright holder for this preprint (which was not certified by peer review) is the author/funder, who has granted bioRxiv a license to display the preprint in perpetuity. It is made available under aCC-BY 4.0 International license.

The transient sodium channel is modelled using parameter values from the Purkinje neuron [1]. This neuron was chosen as a baseline as it contains the same subunits, i.e., NaV1.1-α, NaV1.2-α, and NaV1.6-α [1], as the transient sodium channel in Kiss1^{ARH} neuron [2].

Persistent sodium current:

$$I_{NaP} = g_{NaP} \cdot m_{NaP,\infty}(V_m) \cdot h_{NaP} \cdot (V_m - E_{Na}),$$

g_{NaP} is the maximum conductance; h_{NaP} is the corresponding inactivation gating variable that obeys the following equation:

$$\frac{dh_{NaP}}{dt} = \frac{h_{NaP,\infty}(V_m) - h_{NaP}}{\tau_{h,NaP}};$$

and the steady-state activation and inactivation functions are given by:

$$m_{NaP,\infty}(V_m) = \frac{1}{1 + e^{-(V_m - V_{m,NaP})/k_{m,NaP}}},$$

$$h_{NaT,\infty}(V_m) = \frac{1}{1 + e^{-(V_m - V_{h,NaP})/k_{h,NaP}}}.$$

The above description of the persistent sodium current was taken from a model of the GnRH neuron [3].

A current:

$$I_A = g_A \cdot m_A \cdot h_A \cdot (V_m - E_K)$$

g_{NaP} denotes the maximum conductance; E_K is the potassium reversal potential; and m_A and h_A are the corresponding activation and inactivation gating variables, which are described by the following equations:

$$\frac{dm_A}{dt} = \frac{m_{A,\infty}(V_m) - m_A}{\tau_{m,A}};$$

$$\frac{dh_A}{dt} = \frac{h_{A,\infty}(V_m) - h_A}{\tau_{h,A}}.$$

The steady-state activation and inactivation functions are given by:

$$m_{A,\infty}(V_m) = \frac{1}{1 + e^{-(V_m - V_{m,A})/k_{m,A}}},$$

$$h_{A,\infty}(V_m) = \frac{1}{1 + e^{-(V_m - V_{h,A})/k_{h,A}}}.$$

The model of the A-current was based on Mendonca's model of Kv4 channels [4], as these channels are also found in Kiss1^{ARH} neurons [5].

BK current

$$I_{BK} = g_{BK} \cdot b_{BK,\infty}(V_m, c) \cdot (V_m - E_K),$$

bioRxiv preprint doi: https://doi.org/10.1101/2024.02.20.581121; this version posted September 3, 2024. The copyright holder for this preprint (which was not certified by peer review) is the author/funder, who has granted bioRxiv a license to display the preprint in perpetuity. It is made available under aCC-BY 4.0 International license.

Here, g_{BK} is the maximum conductance; and $b_{BK,\infty}(V_m, c)$ is the steady-state activation function that depends on the membrane potential, V_m, as well as on the cytosolic calcium concertation, c:

$$b_{BK,\infty}(V_m, c) = \frac{1}{1 + e^{-(V_m - V_{BK}(c))/k_{BK}}},$$

$$V_{BK}(c) = V_{BK,0} - k_{shift} \log \frac{c}{k_{c,BK}}.$$

The model of the BK-current was based on the model presented in [6], with the conductance parameter fitted to the current-voltage relationships recorded from Kiss1^{ARH} neurons in the absence and presence of the specific BK blocker, iberiotoxin.

SK current

$$I_{SK} = g_{SK} \cdot b_{SK,\infty}(c) \cdot (V_m - E_K)$$

g_{SK} denotes the maximum conductance; and $b_{SK,\infty}(c)$ is the steady-state activation function, which depends on the cytosolic calcium concertation, c:

$$b_{SK,\infty}(c) = \frac{c^n}{c^n + K_{SK}^n}.$$

The model of the BK-current was based on the model presented in [7], with the conductance fitted to the current-voltage relationships recorded from Kiss1^{ARH} neurons in the absence and presence of the specific SK blocker, apamin.

M current

$$I_M = g_M \cdot m_M \cdot (V_m - E_K)$$

g_M denotes the maximum conductance, and m_M is the corresponding activation gating variable:

$$\frac{dm_M}{dt} = \frac{m_{M,\infty}(V_m) - m_M}{\tau_{m,M}}$$

with the steady-state activation function, $m_{M,\infty}(V_m)$, taking the form:

$$m_{A,\infty}(V_m) = \frac{1}{1 + e^{-(V_m - V_{m,A})/k_{m,A}}}.$$

The model of the M-current was parameterised using the steady-state voltage-clamp measurements from actuate Kiss1 neurons [8], while for the activation timescale we used the timescale used in a model of the CA1/3 pyramidal cells [9].

h current

$$I_h = g_h \cdot [p_h \cdot m_{h,1} + (1 - p_h) \cdot m_{h,2}] \cdot (V_m - E_h),$$

g_h denotes the maximum conductance; and $m_{h,1}$ and $m_{h,2}$ are separate activation gating variables operating on different timescales ($\tau_{m,h,1}$ and $\tau_{m,h,2}$ respectively):

bioRxiv preprint doi: https://doi.org/10.1101/2024.02.20.581121; this version posted September 3, 2024. The copyright holder for this preprint (which was not certified by peer review) is the author/funder, who has granted bioRxiv a license to display the preprint in perpetuity. It is made available under aCC-BY 4.0 International license.

$$\frac{dm_{h,1}}{dt} = \frac{m_{h,1,\infty}(V_m) - m_{h,1}}{\tau_{m,h,1}}$$

$$\frac{dm_{h,2}}{dt} = \frac{m_{h,2,\infty}(V_m) - m_{h,2}}{\tau_{m,h,2}}$$

The corresponding steady-state activation functions are:

$$m_{h,1,\infty}(V_m) = \frac{1}{1 + e^{-(V_m - V_{m,h,1})/k_{m,h,1}}},$$

$$m_{h,2,\infty}(V_m) = \frac{1}{1 + e^{-(V_m - V_{m,h,2})/k_{m,h,2}}}.$$

Finally, parameter p_h dictates the relative contribution of and $m_{h,1}$ and $m_{h,2}$ to the total current.

This model of the h-current is based on the hippocampal CA1 pyramidal neuron [7].

T-type calcium current

$$I_T = g_T \cdot m_{T,\infty} \cdot [p_T \cdot h_{T,1} + (1 - p_T) \cdot h_{T,2}] \cdot (V_m - E_{Ca}),$$

g_T is the maximum conductance; and $h_{T,1}$ and $h_{T,2}$ are separate inactivation gating variables operating on different timescales ($\tau_{h,T,1}$ and $\tau_{h,T,2}$ respectively):

$$\frac{dh_{h,1}}{dt} = \frac{h_{T,1,\infty}(V_m) - h_{T,1}}{\tau_{h,T,1}}$$

$$\frac{dh_{h,2}}{dt} = \frac{h_{T,2,\infty}(V_m) - h_{T,2}}{\tau_{h,T,2}}$$

The corresponding steady-state inactivation functions are:

$$h_{T,1,\infty}(V_m) = \frac{1}{1 + e^{-(V_m - V_{h,T,1})/k_{h,T,1}}},$$

$$h_{T,2,\infty}(V_m) = \frac{1}{1 + e^{-(V_m - V_{h,T,2})/k_{h,T,2}}}.$$

The steady-state activation function is given by:

$$m_{T,\infty} = \frac{1}{1 + e^{-(V_m - V_{m,T})/k_{m,T}}}.$$

Finally, parameter p_T dictates the relative contribution of and $h_{T,1}$ and $h_{T,2}$ to the total current.

To model of the T-current was based on AVPV kisspeptin neurons data presented in [2, 10].

L-, N-, P/Q-, R-type calcium currents

$$I_{Ca} = g_h \cdot m_{Ca} \cdot h_{Ca} \cdot (V_m - E_{Ca})$$

g_{Ca} denotes the maximum conductance; and m_{Ca} and h_{Ca} are the corresponding activation and inactivation gating variables, which are described by the following equations:

bioRxiv preprint doi: https://doi.org/10.1101/2024.02.20.581121; this version posted September 3, 2024. The copyright holder for this preprint (which was not certified by peer review) is the author/funder, who has granted bioRxiv a license to display the preprint in perpetuity. It is made available under aCC-BY 4.0 International license.

$$\frac{dm_{Ca}}{dt} = \frac{m_{Ca,\infty}(V_m) - m_{Ca}}{\tau_{m,Ca}};$$

$$\frac{dh_{Ca}}{dt} = \frac{h_{Ca,\infty}(V_m) - h_{Ca}}{\tau_{h,Ca}}.$$

The steady-state activation and inactivation functions are given by:

$$m_{Ca,\infty}(V_m) = \frac{1}{1 + e^{-(V_m - V_{m,Ca})/k_{m,Ca}}},$$

$$h_{Ca,\infty}(V_m) = \frac{1}{1 + e^{-(V_m - V_{h,Ca})/k_{h,Ca}}}.$$

Parameters of the model for the high voltage activated calcium channels were calibrated from the current-voltage relationships obtained from $Kiss1^{ARH}$ neurons (see Figure 2 and 6 of the main text).

TRPC5 current

$$I_{TRPC5} = g_{TRPC5} \cdot b_{TRPC5}(c, R_{TRPC5,act}) \cdot (V_m - E_{TRPC5})$$

g_{TRPC5} denotes the maximum conductance; and $b_{TRPC5}(c, R_{TRPC5,act})$ the activating gating variable that depends both on cytosolic calcium concertation (c) and on NKB-mediated activation of an intermediary effector, $R_{TRPC5,act}$ [11]:

$$b_{TRPC5}(c, R) = \frac{R_{TRPC5,act}}{1 + e^{-(c - c_{TRPC5})/k_{TRPC5}}}$$

The dynamics of $R_{TRPC5,act}$ (activated form of R_{TRPC5}) are described by:

$$\frac{dR_{TRPC5,act}}{dt} = \left(k_{R_{TRPC5},0} + k_{R_{TRPC5}} \frac{NKB^{n2}}{NKB^{n2} + K_{NKB}^{n2}}\right) \cdot (R_{TRPC5,T} - R_{TRPC5,act}) - k_{-R_{TRPC5}} \cdot R_{TRPC5,act};$$

where NKB is the extracellular NKB concertation; $k_{R,0}$ is the basal rate of R_{TRPC5} activation; k_R is the maximal rate of R_{TRPC5} activation in the presence of NKB; k_{-R} is the rate of R_{TRPC5} inactivation; and $R_{TRPC5,T}$ is the total concentration of the effector.

GIRK current

$$I_{GIRK} = g_{GIRK} \cdot b_{GIRK}(V_m, R_{GIRK,act}) \cdot (V_m - E_K)$$

g_{GIRK} denotes the maximum conductance; and $b_{GIRK}(V_m, R_{GIRK,act}B)$ the activating gating variable that depends on membrane potential, V_m, and on external activation of an intermediary effector, $R_{GIRK,act}$:

$$b_{GIRK}(V_m, R_{GIRK,act}) = m_{GIRK}(V_m)\, R_{GIRK,act}(s)$$

The dynamics of m_{GIRK} are described by:

$$\frac{dm_{GIRK}}{dt} = \frac{m_{GIRK,\infty}(V_m) - m_{GIRK}}{\tau_{GIRK}(V_m)}$$

where the steady state activation function and timescale function are given by:

$$m_{GIRK,\infty}(V_m) = \frac{1}{1 + e^{-\frac{V_m - V_{GIRK}}{k_{GIRK,1}}}} + \frac{\alpha}{1 + e^{-\frac{V_m - V_{GIRK}}{k_{GIRK,2}}}}$$

bioRxiv preprint doi: https://doi.org/10.1101/2024.02.20.581121; this version posted September 3, 2024. The copyright holder for this preprint (which was not certified by peer review) is the author/funder, who has granted bioRxiv a license to display the preprint in perpetuity. It is made available under aCC-BY 4.0 International license.

$$\tau_{GIRK}(V_m) = \frac{1}{\alpha_\tau e^{-(V_m/V_{GIRK})} + \beta_\tau e^{-(V_m/V_{GIRK})}}$$

The dynamics of $R_{GIRK,act}$ are described by:

$$\frac{dR_{GIRK,act}}{dt} = \left(k_{R_{GIRK},0} + k_{R_{GIRK}} \frac{s^{n3}}{s^{n3} + K_s^{n3}}\right) \cdot (R_{GIRK,T} - R_{GIRK,act}) - k_{-R_{GIRK}} \cdot R_{GIRK,act} ;$$

where s is the extracellular concertation of the activation signal.

The model and parameters of the GIRK current is taken from [12].

Leak currents

$$I_{leak} = I_{leak,0} + I_{leak,Ca} = g_{leak,1} \cdot (V_m - E_K) + g_{leak,2} \cdot (V_m - E_{Ca})$$

The leak current parameters were calibrated to current-voltage relationships recorded from $Kiss1^{ARH}$ neurons in the absence/presence of iberiotoxin (BK blocker) and apamin (SK blocker).

Intracellular calcium dynamics

Finally, the intracellular calcium dynamics are described via the following equation:

$$\frac{dc}{dt} = -(I_{Ca} + I_T + I_{TRPC5} + I_{leak,Ca}) \cdot \gamma - d_{ca} \cdot c$$

where parameter γ converts the currents to molecule fluxes and parameter d_{ca} dictates the linear rate at which calcium is depleted or pumped out of the cell.

Modelling the effect of E2

The effect of E2 on ionic currents is modelled as a change in the maximum conductance parameter. For currents I_M, I_T, I_{Ca} and I_{TRPC5} this change is inferred from the qPCR data assuming that the conductance is directly proportional to the mRNA expression. For I_{SK}, I_{BK}, I_{leak}, the OVX and OVX+E2 conductances are obtained from current-voltage relationships recorded from $Kiss1^{ARH}$ neurons in the absence/presence of iberiotoxin (BK blocker) and apamin (SK blocker). All other currents were assumed to be unaffected by E2. Parameter C_m in calibrated using direct measurements of the membrane conductance in the OVX and OVX+E2 state. For the simulation presented in the Figure 13 of the main text, conductances were varied within ranges that captures the physiological effect of E2.

Computer simulations

Integration of the differential equations describing the model was carried out in MATLAB R2023b using a standard 4th order Runge-Kutta method. Parameter fitting was also conducted in MATLAB R2023b using the least squares curve fitting method.

bioRxiv preprint doi: https://doi.org/10.1101/2024.02.20.581121; this version posted September 3, 2024. The copyright holder for this preprint (which was not certified by peer review) is the author/funder, who has granted bioRxiv a license to display the preprint in perpetuity. It is made available under aCC-BY 4.0 International license.

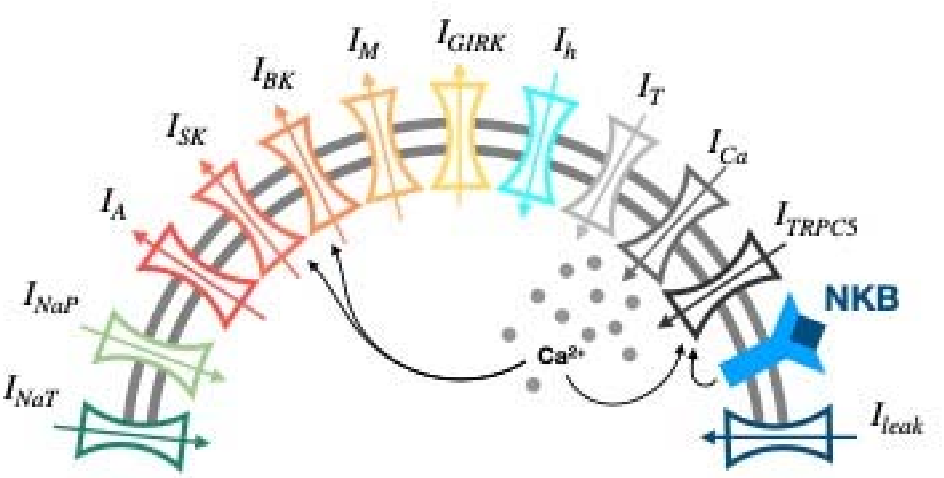

Figure S1. Schematic diagram of the conductance based mathematical model of Arcuate nucleus Kiss1 neurons.

Model parameters					
C_m	23.13 nF (19.5 in OVX+E2)				
Current parameters [1]					References
I_{NaT}	$\tau_{h,NaT}$	4.5 ms	$V_{h,NaT}$	-43.2 mV	[1, 2]
	$V_{m,NaT}$	-25 mV	$k_{m,NaT}$	3 mV	
	$k_{h,NaT}$	-8.2 mV	g_{NaT}	90 nS	
	E_{Na}	66.1 mV			
I_{NaP}	$\tau_{h,NaP}$	250 ms	$V_{h,NaP}$	-47.4 mV	[3]
	$V_{m,NaP}$	-41.5 mV	$k_{m,NaP}$	3 mV	
	$k_{h,NaP}$	-8.5 mV	g_{NaP}	3.37 nS	
	E_{Na}	66.1 mV			
I_A	$\tau_{h,A}$	10 ms	$V_{h,A}$	-55.1 mV	[4, 5]
	$\tau_{m,A}$	20 ms	$V_{m,A}$	-30 mV	
	$k_{h,A}$	-11.4 mV	$k_{m,A}$	10 mV	

[1] Parameters $g_{\cdot}$ denote the maximum conductance associated with each current.

bioRxiv preprint doi: https://doi.org/10.1101/2024.02.20.581121; this version posted September 3, 2024. The copyright holder for this preprint (which was not certified by peer review) is the author/funder, who has granted bioRxiv a license to display the preprint in perpetuity. It is made available under aCC-BY 4.0 International license.

	g_A	60 nS in OVX<br>35 in OVX+E2	E_K	-81 mV	
I_{BK}	k_{BK}	10 mV	k_{shift}	18 mV	[6]
	$V_{BK,0}$	-22.52 mV	$k_{c,BK}$	1.5 µM	
	g_{BK}	13.50 nS in OVX;<br>20 nS in OVX+E2	E_K	-81 mV	
I_{SK}	K_{SK}	0.45 µM	n	4	[13]
	g_{SK}	28.13 nS in OVX;<br>26.05 nS in OVX+E2	E_K	-81 mV	
I_M	$\tau_{m,M}$	10 ms	$V_{m,M}$	-50 mV	[8, 9]
	$k_{m,M}$	20 mV	g_M	0.23 nS in OVX<br>1.23 ns in OVX+E2	
	E_K	-81 mV			
I_h	$\tau_{m,h,1}$	80 ms	$V_{m,h,1}$	-102 mV	[7, 14, 15]
	$\tau_{m,h,1}$	310 ms	$V_{m,h,2}$	-102 mV	
	p_h	0.85	$k_{m,h,1}$	-10 mV	
	$k_{m,h,2}$	-10 mV	g_h	0.56 nS (11.23 nS in OVX+E2)	
	E_h	-27.8 mV			
I_T	$\tau_{h,T,1}$	1.94 ms	$V_{h,T,1}$	-69.1 mV	[2, 10]
	$\tau_{h,T,2}$	86.3 ms	$V_{h,T,2}$	-69.1 mV	
	p_h	0.5	$V_{m,T}$	-54 mV	
	$k_{h,T,1}$	-5.3 mV	$k_{h,T,2}$	-5.3 mV	
	$k_{m,T}$	3.3 mV	g_T	0.66 nS (5 nS in OVX+E2)	
I_{Ca}	$\tau_{h,Ca}$	10 ms	$V_{h,Ca}$	-48.9	fitted
	$\tau_{m,Ca}$	300 ms	$V_{m,Ca}$	-27.3 mV	

bioRxiv preprint doi: https://doi.org/10.1101/2024.02.20.581121; this version posted September 3, 2024. The copyright holder for this preprint (which was not certified by peer review) is the author/funder, who has granted bioRxiv a license to display the preprint in perpetuity. It is made available under aCC-BY 4.0 International license.

	$k_{h,Ca}$	-18.2	$k_{m,Ca}$	4.62 mV	
	g_{Ca}	2.1 nS (2.8 nS in OVX+E2)	E_{Ca}	121.6 mV	
I_{TRPC5}	c_{TRPC5}	0.6 μM	k_{TRPC5}	0.33 μM	[11]
	$k_{R_{TRPC5}}$	0.006 ms^{-1}	$k_{-R_{TRPC5}}$	0.002 ms^{-1}	
	$k_{R_{TRPC5},0}$	0.002 ms^{-1}	g_{TRPC5}	8.4 nS (1.68 nS in OVX+E2)	
	K_{NKB}	32 μM	$n2$	2	
	$R_{TRPC5,T}$	1 μM	E_{TRPC5}	-15 mV	
I_{GIRK}	V_{GIRK}	-70 mV	$k_{GIRK,1}$	20 mV	[12]
	α	0.8	α_{τ}	0.0061	
	$k_{GIRK,2}$	100 mV	β_{τ}	0.0818	
	g_{GIRK}	0.4 nS	E_K	-81 mV	
	$k_{R_{GIRK},0}$	0 ms^{-1}	$k_{R_{GIRK}}$	10^{-3} ms^{-1}	
	$R_{GIRK,T}$	1 μM	K_S	1 μM	
	$k_{-R_{GIRK}}$	3.3·10^{-6} ms^{-1}	$n3$	2	
I_{leak}	$g_{leak,1}$	3.5 nS in OVX<br>3.65 nS in OVX+E2	$g_{leak,2}$	4 nS	fitted
Intracellular calcium					
	γ	1.96 ·10^{-5} μM nA^{-1} ms^{-1}	d_{ca}	0.008 ms^{-1}	[6]

Table S1 Table of model parameters.

References

bioRxiv preprint doi: https://doi.org/10.1101/2024.02.20.581121; this version posted September 3, 2024. The copyright holder for this preprint (which was not certified by peer review) is the author/funder, who has granted bioRxiv a license to display the preprint in perpetuity. It is made available under aCC-BY 4.0 International license.

1. Fry, M., Boegle, A.K. and Maue, R.A. 2007. Differentiated pattern of sodium channel expression in dissociated Purkinje neurons maintained in long-term culture. *Journal of Neurochemistry* **101**:737-748. PMID: 17448145.

2. Zhang, C., Bosch, M.A., Qiu, J., Ronnekleiv, O.K. and Kelly, M.J. 2015. 17β-Estradiol increases persistent Na^+ current and excitability of AVPV/PeN Kiss1 neurons in female mice. *Molecular Endocrinology* **29**:518-527. PMID: 25734516.

3. Moran, S., Moenter, S.M. and Khadra, A. 2016, A unified model for two modes of bursting in GnRH neurons. *Journal of Computational Neuroscience* **40**, 297-315. PMID: 26975615.

4. Mendonça, P.R., Vargas-Caballero, M., Erdelyi, F., Szabo, G., Paulsen, O. and Robinson, H.P. 2016. Stochastic and deterministic dynamics of intrinsically irregular firing in cortical inhibitory interneurons. *eLife* **5**:e16475. PMID: 27536875.

5. Mendonça, P.R., Kyle, V., Yeo, S.H., Colledge, W.H. & Robinson, H.P. 2018. Kv4. 2 channel activity controls intrinsic firing dynamics of arcuate kisspeptin neurons. *The Journal of Physiology* **596**:885-899. PMID: 29214635.

6. Tsaneva-Atanasova, K., Sherman, A., Goor, F.v. and Stojilkovic, S.S. 2007. Mechanism of spontaneous and receptor-controlled electrical activity in pituitary somatotrophs: experiments and theory. *Journal of Neurophysiology* **98**:131-144. PMID: 17493919.

7. Booth, C.A., Witton, J., Nowacki, J., Tsaneva-Atanasova, K., Jones, M.W., Randall, A.D., Brown, J.T. 2016. Altered intrinsic pyramidal neuron properties and pathway-specific synaptic dysfunction underlie aberrant hippocampal network function in a mouse model of tauopathy. *Journal of Neuroscience* **13**:350-63. PMID: 26758828.

8. Conde, K. and Roepke, T. 2020. 17β-estradiol increases arcuate KNDy neuronal sensitivity to ghrelin inhibition of the M-current in female mice. *Neuroendocrinology* **110**:582-594. PMID: 31484184.

9. Nowacki, J., Osinga, H.M., Brown, J.T., Randall, A.D. & Tsaneva-Atanasova, K. 2011. A unified model of CA1/3 pyramidal cells: An investigation into excitability. *Progress in Biophysics and Molecular Biology* **105**:34-48. PMID: 20887748.

10. Wang, L., DeFazio, R.A. and Moenter, S.M. 2016. Excitability and burst generation of AVPV kisspeptin neurons are regulated by the estrous cycle via multiple conductances modulated by estradiol action. *eNeuro* **3**, e0094-0016.2016. PMID: 27280155.

11. Qiu, J., Stincic, T.L., Bosch, M.A., Connors, A.M., Petrie, S.K., Rønnekleiv, O.K. and Kelly, M.J. 2021. Deletion of Stim1 in hypothalamic arcuate nucleus Kiss1 neurons potentiates synchronous GCaMP activity and protects against diet-induced obesity. *Journal of Neuroscience* **41**:9688-9701. PMID: 34654752.

12. Tian, J.-b., Yang, J., Joslin, W.C., Flockerzi, V., Prescott, S.A., Birnbaumer, L. and Zhu, M.X. 2022.TRPC4 and GIRK channels underlie neuronal coding of firing patterns that reflect $G_{q/11}$ - $G_{i/o}$ coincidence signals of variable strengths. *Proceedings of the National Academy of Sciences* **119**:e2120870119. PMID: 35544691

13. Bond, C.T., Maylie, J., Adelman, J.P. 1999. Small-conductance calcium-activated potassium channels. *Annals of New York Acadamy of Sciences*. **868**:370-8. PMID: 10414306.

14. Gottsch, M.L., Popa, S.M., Lawhorn, J.K., Qiu, J., Tonsfeldt, K.J., Bosch, M.A., Kelly, M.J., Rønnekleiv, O.K., Sanz, E., McKnight, G.S., Clifton, D.K., Palmiter, R.D. and Steiner, R.A. 2011. Molecular properties of Kiss1 neurons in the arcuate nucleus of the mouse. *Endocrinology* **152**:4298-4309. PMID: 21933870.

bioRxiv preprint doi: https://doi.org/10.1101/2024.02.20.581121; this version posted September 3, 2024. The copyright holder for this preprint (which was not certified by peer review) is the author/funder, who has granted bioRxiv a license to display the preprint in perpetuity. It is made available under aCC-BY 4.0 International license.

15. Qiu, J., Rivera, H.M., Bosch, M.A., Padilla, S.L., Stincic, T.L., Palmiter, R.D., Kelly, M.J. and Rønnekleiv, O.K. 2018. Estrogenic-dependent glutamatergic neurotransmission from kisspeptin neurons governs feeding circuits in females. *eLife* **7**:e35656. PMID: 30079889.
